# Immune-cancer interactions in tumors and tumor-draining lymph nodes: Novel prognostic indicators for breast cancer

**DOI:** 10.1186/2051-1426-2-S3-P255

**Published:** 2014-11-06

**Authors:** James Mansfield, Peter Lee

**Affiliations:** 1 PerkinElmer, Hopkinton, MA, USA; 2 City of Hope Comprehensive Cancer Center, CA, USA

## 

It is becoming clear that immune cells play many important but sometimes conflicting roles in cancer. Immune profile changes at sites of immune-cancer interactions, such as the tumor microenvironment and tumor-draining lymph nodes (TDLNs), may represent a sensitive predictor of local and distant tumor metastasis. However, standard pathologic analysis of tumor sections has remained at the visual assessment of one marker per serial section level; it would be extremely useful to be able to visualize the distributions of multiple phenotyped immune and other cells in-situ in solid tumors to dissect the complex interplay between immune/stromal cells and cancer cells within tumors, tumor-draining lymph nodes (TDLNs), and blood. We generate immune profiles that include complete immunophenotyping and identification of cellular spatial relationships within and between the tumor microenvironment and TDLNs from formalin-fixed paraffin-embedded lymph node and tumor specimens from cancer patients using a combination of multiplexed IHC/IF, multispectral imaging, and automated image analysis which delivers quantitative per-cell measures of each marker. These per-cell intensities are then translated into a phenotype for each cell. We have found that immune cell populations as well as their spatial distributions and clustering patterns have strong correlation with clinical outcome.

